# A Physical Map of the Short Arm of Wheat Chromosome 1A

**DOI:** 10.1371/journal.pone.0080272

**Published:** 2013-11-21

**Authors:** James Breen, Thomas Wicker, Margarita Shatalina, Zeev Frenkel, Isabelle Bertin, Romain Philippe, Wolfgang Spielmeyer, Hana Šimková, Jan Šafář, Federica Cattonaro, Simone Scalabrin, Federica Magni, Sonia Vautrin, Hélène Bergès, International Wheat Genome Sequencing Consortium, Etienne Paux, Tzion Fahima, Jaroslav Doležel, Abraham Korol, Catherine Feuillet, Beat Keller

**Affiliations:** 1 Institute of Plant Biology, University of Zurich, Zurich, Switzerland; 2 Institute of Evolution, University of Haifa, Haifa, Israel; 3 INRA UMR 1095, Genetique Diversite et Ecophysiologie des Cereales, Clermont-Ferrand, France; 4 CSIRO Plant Industry, Canberra, Australia; 5 Centre of the Region Hana for Biotechnological and Agricultural Research, Institute of Experimental Botany, Olomouc, Czech Republic; 6 Istituto di Genomica Applicata, Udine, Italy; 7 CNRGV - INRA, Castanet Tolosan, France; Cankiri Karatekin University, Turkey

## Abstract

Bread wheat (*Triticum aestivum*) has a large and highly repetitive genome which poses major technical challenges for its study. To aid map-based cloning and future genome sequencing projects, we constructed a BAC-based physical map of the short arm of wheat chromosome 1A (1AS). From the assembly of 25,918 high information content (HICF) fingerprints from a 1AS-specific BAC library, 715 physical contigs were produced that cover almost 99% of the estimated size of the chromosome arm. The 3,414 BAC clones constituting the minimum tiling path were end-sequenced. Using a gene microarray containing ∼40 K NCBI UniGene EST clusters, PCR marker screening and BAC end sequences, we arranged 160 physical contigs (97 Mb or 35.3% of the chromosome arm) in a virtual order based on synteny with *Brachypodium*, rice and sorghum. BAC end sequences and information from microarray hybridisation was used to anchor 3.8 Mbp of Illumina sequences from flow-sorted chromosome 1AS to BAC contigs. Comparison of genetic and synteny-based physical maps indicated that ∼50% of all genetic recombination is confined to 14% of the physical length of the chromosome arm in the distal region. The 1AS physical map provides a framework for future genetic mapping projects as well as the basis for complete sequencing of chromosome arm 1AS.

## Introduction

The Poaceae family of grass species includes some of the most economically important agricultural crop species in the world. Plants such as rice (*Oryza sativa*), sorghum (*Sorghum bicolor*), maize (*Zea mays*), wheat (*Triticum aestivum*) and barley (*Hordeum vulgare*) provide a large share of the world’s food. In order to increase crop productivity, genome sequencing projects have been initiated to provide genetic tools for plant improvement [1]. Grass species such as rice [2], sorghum [3], *Brachypodium distachyon* [4] have had their genomes completely sequenced. For barley, a partial genome sequence became recently available which covers gene space but is incomplete in repetitive intergenic regions [5].

Model genomes such as rice [2], sorghum [3] and *Brachypodium* [5] have been used to infer gene order in wheat and barley: positional information of genes from syntenic regions in these three model genomes was compiled for the production of “chromosome zippers” [6]. These were used to deduce a virtual gene order of the barley genome [7] and wheat group 1 chromosomes [8]. However, these studies as well as sequencing of large contigs of chromosome 3B [9] also showed that both wheat and barley contain approximately 50% of non-colinear genes which cannot be ordered based on synteny to model genomes. Thus, the production of physical maps and ultimately a complete genome sequence are essential for map-based cloning and to determine the correct order of genes. In wheat however, genome sequencing efforts are hampered by a large 17,000 Mb genome size (over 43 times larger than the rice genome), >80% repetitive sequence [10], [11] and hexaploid (AABBDD) genome structure.

One of the most important resources for physical mapping are bacterial artificial chromosome (BAC) libraries. Whole genome sequencing projects of Arabidopsis [12], rice [2] and maize [4], [13], [14] have all benefited from BAC-based physical maps to produce a scaffold structure for the construction of large, assembled chromosome sequences or pseudomolecules. Contigs constructed from BAC clones provide a backbone to which sequence contigs can be anchored. This is especially valuable in large and repetitive genomes where simple whole-genome shotgun sequencing usually leads large numbers of un-ordered sequence contigs.

To reduce complexity of the wheat genome analysis, individual chromosomes (for the largest chromosome 3B) or chromosome arms can be isolated through cytometric flow-sorting based on their individual ‘flow karyotype’ [15]. Ditelosomic chromosome-specific wheat lines are used to purify individual chromosome arms that can then be used for the production of chromosome-specific BAC libraries, survey sequencing and physical maps [9], [15], [16], [17]. Chromosome 3B, the largest chromosome in wheat (∼1 Gb), was the first chromosome to be physically mapped. A chromosome 3B-specific BAC library was produced from flow-sorted chromosomes which was then used for the development of the physical map of wheat chromosome 3B [18].

Flow-sorted chromosome arms were used recently for shotgun sequencing with Illumina technology in the framework of the International Wheat Genome Sequencing Consortium (IWGSC). The sequence assemblies of this so-called “whole chromosome survey sequencing” are available through the IWGSC website (wheatgenome.org). The assemblies resulted in hundreds of thousands of sequence contigs per chromosome arm. These sequence contigs are mostly un-ordered but, nevertheless, are expected to cover almost the complete gene space of the individual chromosome arms. A fraction of these sequences was ordered based on synteny using the chromosome zipper method [6]. Furthermore, the entire genome of wheat was recently surveyed by whole-genome shotgun sequencing. However, due to the large size and high repeat content of the genome, sequence assembly had to be limited on the gene-containing fraction [19].

BAC clones are typically digested with restriction enzymes which produces a specific fragment pattern for each BAC clone. This “fingerprint” is then used to find overlaps between BACs and arrange them into physical BAC contigs. Modern high information content fingerprinting (HICF) is currently using restriction enzymes that produce particularly diverse fingerprint patterns. FPC [20] has been the most popular fingerprint construction program for previous grass genome projects. However, the application of FPC is limited when dealing with complex genomes such as wheat because highly repetitive, chimeric or poorly fingerprinted “questionable” clones (Q-clones) can create false overlaps and thus wrongly assemble contigs (called Q-contigs [21]). To improve fingerprint contig assembly in highly repetitive genomes, the Linear Topological Contig (LTC) algorithm [21] was developed. It improves contig assembly by avoiding large branching structures from multiple neighbouring BAC connections, to create physical contigs with linear topology.

The available genomic sequence resources in wheat described above are complemented by over 1 million expressed sequence tags (ESTs) which are publicly available at NCBI (ncbi.nlm.nih.gov). These allow the characterisation of expressed genes as well as the assembly of so-called UniGene EST contigs. For wheat, a NimbleGen 12×135 K microarray was designed containing 40,349 probes made from wheat UniGene sequences [22]. This microarray can be used as a proxy to associate gene content with BAC clones without having the complete sequence information: hybridising BAC pools to that microarray allows the rapid identification of BAC clones containing a homolog of a UniGene that is present on the microarray. However, the results have to be used with caution because hybridisation signals do not distinguish between orthologs, paralogs, pseudogenes or gene fragments. Nevertheless, this resource was successfully used for the construction of a 3,000-locus transcriptome map for wheat chromosome 3B [22].

Low resolution physical mapping of genes has been done for several years on wheat lines which contain partial deletions of chromosome arms. This so-called “deletion-bin mapping” was done with wheat ESTs across all wheat chromosomes [23], [24] and showed that genes were more frequently found in distal chromosomal regions than in proximal regions. Recent studies using transcriptome mapping [22], [25] largely confirmed these findings but also indicated that about 75% of the genes on chromosome 3B are located in small gene islands containing on average three genes [25], thereby supporting earlier studies that suggested clustering of genes in certain regions [26], [27].

The short arm of chromosome 1A is one of the smallest chromosome arms of wheat and was estimated through cytogenetic methods to have a size of 275 Mb [15]. Chromosome arm 1AS is free of large C-banding regions that indicate regions of constitutive heterochromatin [28]. It contains the disease resistance genes *Pm3* [29] and *Lr10* [30] as well as gene underlying other important traits such as low molecular weight (LMW) glutenin genes [31] and the tiller inhibition gene (*tin*) [32]. Furthermore, it contains at least three families of resistance gene analogs (RGAs, [33]).

As member of the International Wheat Genome Sequencing Consortium, we have produced a physical map of the short-arm of chromosome 1A. This physical map was assembled through high-information content fingerprinting (HICF) of a chromosome 1AS-specific BAC library using two different physical mapping algorithms and anchored to a chromosome 1AS transcriptome map using high-throughput microarray hybridisation. Additionally, we produced an integrated sequence model that consists of genomic sequences that were ordered based on all available information resources. It comprises at total of 3.8 Mbp of sequences and consists of BAC-end sequences and 1AS survey sequence contigs that were anchored to the BAC contigs through genetic mapping and NimbleGen hybridisation data. This highly integrated map can serve as a framework for future sequencing and map-based cloning studies.

## Methods

### Purification of Chromosome Arm 1AS

Aqueous suspensions of metaphase chromosomes were prepared from root tips of double ditelosomic line (2n = 40+2t1AS+2t1AL) of *Triticum aestivum* L. cv. ‘Chinese Spring’ according to Vrána et al. [34]. After staining by DAPI, the samples were analysed using FACSVantage flow sorter and 1AS arm was purified. The arm was sorted in batches of 200,000 into 320 µl of 1.5×IB buffer [35], and a total of 8.85 million 1AS arms were collected. The identity and purity in sorted fractions was checked by FISH using probes for telomeric repeats and GAA repeats [36]. The purity of the sorted 1As fraction was 94% (olomouc.ueb.cas.cz/dnalib/taacsp1asha), and 6% of the collected DNA stemmed from other wheat chromosomes.

### Construction of 1AS-specific BAC Library

The library (named TaaCsp1AShA) was constructed as previously described [37]. High molecular weight DNA was partially digested with *Hin*dIII (New England Biolabs, Beverly, USA) and subjected to two rounds of size selection. DNA of particular size fractions was electro-eluted from the gel and ligated into pIndigoBAC-5 vector (Epicentre, Madison, USA). The recombinant vector was used to transform *Escherichia coli* DH10B competent cells (Invitrogen, Carlsbad, USA). The library was ordered into 384-well plates filled with freezing medium consisting of 2YT, 6.6% glycerol and 12.5 µg/ml Chloramphenicol and stored at −80°C. In order to estimate average insert size, 160 BAC clones were randomly selected from both size fractions of the library and analyzed as previously described [36].

### Fingerprint Assembly and Minimum Tiling Path (MTP) Selection

Fingerprinting of the chromosome 1AS-specific BAC library [15] was performed using a modified SNaPshot high-information content fingerprinting (HICF [38]) protocol used previously for creating hexaploid wheat physical maps [18]. Fingerprint background (vector, low signal and partially digested-related peaks) was removed using FPB [39] and contamination was removed using GenoProfiler [40].

Automated computational assembly of fingerprints was performed using FPC [19]. Standard physical map assembly procedures were followed based on previously described protocols from wheat physical mapping [18]: Initial assembly of 25,918 clones (incremental contig building) was performed at 1E-75, then merging (single-to-end followed by end-to-end) was performed step-wise by increasing the cut-off (1E-5 at each step) to a final cut-off of 1E-45. The DQer function was used after each merge to break up all contigs that contained more than 10% of questionable (Q) clones. The minimum tiling path (MTP) was selected with the FPC MTP module [41], which was then used to create three-dimensional pools for marker screening [42] and BAC-end sequencing using Sanger ABI3730 sequencer. The 3D-pools were validated using the published chromosome 1AS cv. Chinese Spring-specific gene markers for *Pm3* [29] and Lr10 [30].

Physical contigs and MTP BACs were screened using LTC [21] to identify problematic contigs, followed by a complete reassembly by LTC. Initial assembly was performed at a stringency of 1E-15, with Q-contigs (contigs displaying non-linear network structures) being separated at higher stringency (up to 1E-45). Network files of all Q-contigs were analysed using Pajek (vlado.fmf.uni-lj.si/pub/networks/pajek/). Q-clones creating non-linear conformations were then fragmented and successive adaptive clustering algorithms were used to produce large contigs. Physical contigs containing at least 6 clones were kept for downstream analysis.

### Sequence Analysis

BAC-end sequences were analysed through BLASTN and BLASTX searches against sequence databases. Triticeae-specific repetitive elements were searched against both nucleotide and protein subsets of the Triticeae-specific repeat database (TREP, wheat.pw.usda.gov/ITMI/Repeats/) to identify characterised transposable elements (TEs). Genes were identified by searching the *Brachypodium distachyon* (IBI2010) gene and tRNA, rDNA, mitochondrial and chloroplast DNA sequences (ncbi.nlm.nih.gov/Genome). Closest *Brachypodium* homologs of Unigene sequences were identified by BLASTX against *Brachypodium* proteins (IBI2010). Hybridised Unigene probe matches were screened for TEs against TREP and searched against deletion bin-mapped 1AS-specific wheat ESTs [43]. All BLASTX searches were performed using BLASTX alignments with a <1E-10 E-value cut-off. Cut-off for BLASTN searches were alignments of at least 50 bp with >80% identity and an E-value of <1E-10.

### Anchoring of Genetic Markers

Genetic markers (Table 1) were gathered from published genetic maps contained in the marker repository GrainGenes (wheat.pw.usda.gov) based on their presence on chromosome 1AS. Initial anchoring of physical contigs to maps was performed using standard PCR screening on MTP 3D-pools using published genetic markers (RFLP and SSRs). Additionally, EST/cDNA-based markers were designed based on low coverage 454 sequencing from flow-sorted chromosome 1AS [9]. If marker sequence data (e.g. primers or RFLP sequences) were available, these sequences were used in Blastn searches against BAC-end and anchored Illumina sequences (see below). For primers, we required perfect matches for anchoring, while we accepted matches with >97% for RFLP sequences.

**Table 1:** Genetic markers linked to the chromosome 1AS physical map.

Marker	Zipper^1^	TmGxG^2^	TaBxT^3^	TaAxF^4^	TaNxW^5^	TaSxO^6^	TaCxCS^7^	ConSSR^8^	Com2004^9^	ConsPos^10^
bcd1434	18	5.1	–	–	–	–	–	–	–	5
psp2999(Pm3)	28	4.8	3	1	–	14	–	–	12	7
whs179	29	–	–	–	–	–	–	–	–	10
gmw136	29	–	–	–	–	–	–	–	14	14
cfa2153	39	–	–	1	-	13.8	5.4	–	15	9
barc148	48	–	–	–	55	33.4	–	56.8	–	48
F640(Lr10)	56	–	–	–	–	–	–	–	–	22
mwg2245b (Lr10)	56	–	–	–	–	–	–	22	–	22
mag1884	88	–	–	–	22	–	–	–	–	22
gpw2005	122	–	–	–	-	–	27.3	–	31	29
wmc24	176	–	–	–	45	35	–	48.8	37	41
gpw2142	343	–	–	–	–	43.1	–	–	54	49
cfd58	452	–	–	52.9	–	–	–	–	-	53
psp3027	496	–	–	–	–	–	–	–	50	50
fba393	588	–	–	–	–	–	12.5	–	21	17
wmc286	C	–	–	–	–	–	–	–	45	45

1 Position of the closest gene in the reference zipper.

2 TmGxG: T. monococcum G1777×G2528 [67].

3 TaBxT: Banks×Banks+tin (Spielmeyer et al., unpublished).

4 TaAxF: Arina×Forno [68].

5 TaNxW: Nanda2419×Wangshuibai [69].

6 TaSxO : Syntethic×Opata [70].

7 TaCxCS: Courtot×Chinese Spring (Sourdille, unpublished).

8 ConSSR: Consensus SSR [53].

9 Com2004: Wheat-Composite 2004 (Appels et al., unpublished).

10 ConsPos: Consensus position (approximate average of all cM positions for each marker).

Genetic positions (telomere to centromere) from different maps were taken from published genetic maps in GrainGenes (wheat.pw.usda.gov). The numbers in the individual marker fields indicate the cM position of the respective marker. Note that markers mapping to the same position in the reference zipper can have multiple different cM positions, depending on the map/population.

### Wheat NimbleGen 40 K UniGene Microarray Hybridisation

Forty-nine DNA pools were generated from the MTP clones. The MTP was derived from FPC contigs which cover approximately 86% of chromosome arm 1AS (see results and discusison). The DNA pools were used for hybridisation on a custom 12×135 K NimbleGen gene expression array (nimblegen.om/produts/expression/index.html) containing 40,349 NCBI wheat UniGene EST clusters (ncbi.nlm.nih.gov/unigene). Hybridisation procedures were carried out as previously described [22] using identical 12×135 K array specifications, but no dye-swap was performed. Normalisation of hybridisation data was performed using the R statistical package (r-project.org). Thresholds from single-dye hybridisation were calculated from the probe data against each pool to eliminate outliers. Genes hybridising to 3 probes were selected and median values were calculated for each gene. Genes with a ratio equal to 1 were eliminated. Box-plots were calculated for all other ratios to identify outliers and define threshold values. These threshold values were then used to remove outlier values for pools labelled with either cy3 or cy5 dyes. Values greater than the mean (+/− standard deviation) for each gene and pool were extracted with previously identified thresholds [22]. To detect positive signals, two different methods were used, the so-called Mean+x Standard deviation (m+xSD) method and t-tests, as described previously [22], [25]. After data de-convolution, unique addresses were taken from the highest stringency of each method (2.4 for plates, 2.7 for rows, 2.8 for columns for M+xSD and p-value 0.01 for t-tests).

Hybridised UniGene probe matches were screened for TEs by Blast against the TREP database. They were also searched against deletion bin-mapped 1AS-specific wheat ESTs [38]. All Blastx searches were performed using a <1E-10 E-value cut-off. BLASTN searches were run using a 50 nucleotide alignment with >80% identity and a <1E-10 E-value cut-off.

### Production of an Integrated Sequence Model from Illumina Survey Sequences

Illumina sequence contigs for chromosome 1AS were obtained though the sequence repository of the International Wheat Genome Sequencing Consortium (wheat-urgi.versailles.inra.fr/Seq-Repository). The 1AS survey sequence assembly consists of 187,490 contigs with a cumulative length of 178 Mbp. To anchor the sequence contigs to the physical BAC contigs, the Illumina contigs were used as queries in Blastn searches against BAC-end sequences of the 1AS minimum tiling path. Blast alignments of >500 bp and >98% sequence identity were accepted for anchoring a sequence. The Illumina contigs were used without repeat masking. This was done to allow anchoring of possible gene sequences that are located next to repetitive sequences on an Illumina contig. The stringent criteria (>500 bp and >98% sequence identity) used for the selection of Blast hits reduces the chance that repetitive sequences are wrongly anchored.

Additionally, Illumina contigs were used in Blast searches against the 40,349 UniGenes that are represented on the NimbleGen microarray. Here, we used a cut-off of 200 bp and >90% sequence identity to anchor a sequence. We used less stringent criteria for UniGenes because the microarray represents genes from the entire wheat genome, which makes it very likely, that many signals come from paralogous genes. We considered the low stringency legitimate because the Illumina survey sequences specifically represent chromosome 1AS, thereby reducing the probability that paralogs were wrongly anchored. However, this does not exclude the possibility that members of large gene families from within 1AS are anchored incorrectly. Each Illumina contig was allowed to be anchored only in one place (i.e. the same sequence can not occur multiple times in the integrated sequence model).

### Data Deposition

The chromosome 1AS BAC library is available through the Centre National de Resources Génomiques Végétales (CNRGV), Toulouse, France (cnrgv.toulouse.inra.fr/) and the Institute of Experimental Botany, Olomouc, Czech Republic (http://olomouc.ueb.cas.cz/dna-libraries/cereals). Physical mapping and marker data is available from the Unité de Recherche Génomique Info (URGI) at Institut National de la Recherche Agronomique (INRA), France (wheat-urgi.versailles.inra.fr/Projects/TriticeaeGenome2). The LTC BAC contig information can be accessed through the dedicated Gbrowse browser.(http://urgi.versailles.inra.fr/gb2/gbrowse/wheat_phys_pub/). The integrated sequence model was deposited at at the URGI sequence repository (http://wheat-urgi.versailles.inra.fr/Seq-Repository) in the 1A section. The sequence can also be obtained from the authors via FTP upon request.

## Results and Discussion

### Chromosome 1AS BAC Fingerprinting and Selection of a Minimum Tiling Path

The 1AS-specific library (TaaCsp1AShA) consists of 31,104 clones with an average insert size of 111 kb. Assuming a cytogenetically estimated chromosome arm size of 275 Mb [15], the library provides a chromosome arm coverage of approximately 11.8x. High information content fingerprinting (HICF, [41], [43]) yielded 25,918 high-quality fingerprints (83.3% of the library). Initial fingerprint assembly using FPC resulted in 805 physical contigs with an average size of 294 kb. The largest contig has a size of approximately 2,114 kb and is comprised of 211 BACs. The total assembly length is 236 Mb corresponding to approximately 86% of the size of 1AS (Table 2). A minimum tiling path (MTP, the minimum number of BAC clones necessary to cover all assembled contigs) of 3,414 BACs was selected from the 805 FPC contigs (average of 4.25 BACs per contig). From this selected MTP, we designed 49 three-dimensional pools (plate, row and column) to allow efficient PCR screening. Additionally, the ends of all MTP BACs were sequenced (see below).

**Table 2:** Comparison of the results of two physical map assembly algorithms used to assemble chromosome 1AS fingerprints, FPC and LTC.

	FPC	LTC
Number of contigs	805	505
Contigs with more than 5 BACs	631	394
Assembly length [Mb]	236	226
Chromosome arm fraction	85.8%	82%
Maximum clones on a contig	274	317
Clones in contigs	20,705	21,622
Singletons	5,213	4,296
Contigs in N50	175	90
Length of N50 contig [kb]	466	798

### BAC Fingerprint Assembly Improvement using the Linear Topological Contig Algorithm

**Figure 1:**
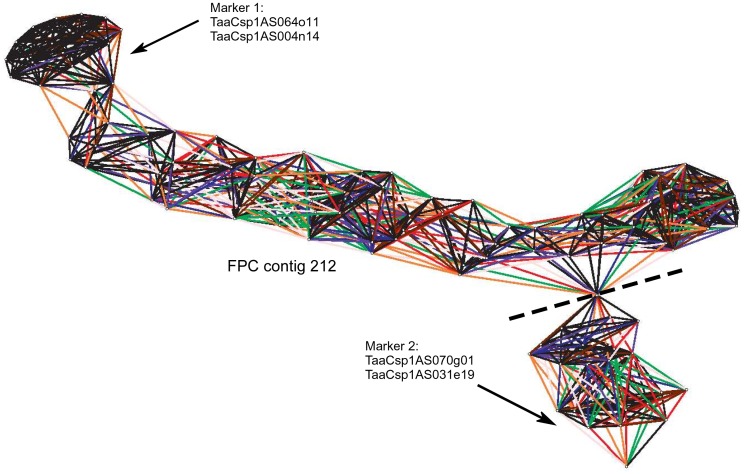
Pajek analysis of BAC contigs from chromosome 1AS assembled with the FPC software. Repetitive BAC clones within the BAC fingerprints were problematic for the FPC assembly, leading to non-linear contig patterns. The reassembly of fingerprints using the LTC assembly program resolved non-linear contigs. The dashed line indicates where the non-linear contig was cut into two contig segments, because the two segments are only connected by a single BAC clone (indicated by all connections converging in one point.

The Linear Topological Contig (LTC) algorithm [21] became available after our initial FPC fingerprinting and MTP selection. Thus, we reassembled fingerprints with LTC to evaluate the accuracy of the FPC protocol. LTC was used to perform clone network analysis of FPC contigs and identify problematic contigs that did not conform to a linear topography (FPC_ctg212 is shown as an example in Figure 1). An initial assembly build on all 25,918 clones was run using LTC to establish physical contigs at a Sulston score of 1E-15 using adaptive clustering methods. 340 physical contigs were constructed and Q-contigs were identified that contained non-linear conformations or Q-clones that were not supported by other, parallel clones (i.e. putative chimeric clones). Many Q-clones causing false connections between clones in Q-contigs were identified to be from the same fingerprint sequencing plate, indicating that well-to-well contamination was a major cause of creating non-linear assembled contigs. Similar problems were previously reported when assembling a FPC map in maize [44]. Q-contigs were then rebuilt with an increased stringency up to 1E-50 in order to disperse the non-linear clusters. After breaking up Q-contigs, the LTC assembly consisted of a total of 505 contigs (Table 2). After all contigs showed linear clone patterns, small contigs containing five clones or less and no marker information (see below) were removed from the assembly as they did not provide any more meaningful information. This resulted in a more accurate final dataset of 394 LTC contigs which was used for subsequent analyses (Table 2).

### Comparison of FPC and LTC Assemblies

The characteristics of the 394 LTC linear contigs were compared to those of the 805 contigs produced with FPC (Table 3). Overall, the LTC assembly included more BAC clones than the FPC assembly (21,622 vs. 20,705), leaving only 4,296 singletons compared to the 5,213 singletons left by FPC. The N50 number of contigs (the least number of contigs that contain 50% of the total assembly) fell from 175 to 90 and the length of the N50 contig increased from 479 kb to 798 kb using LTC (Table 3).

**Table 3:** Final composition of the BAC clone backbone of the wheat 1AS physical map.

LTC contigs with >5 BACs	394
BACs in LTC contigs	21,255
Total size of LTC contigs [Mbp]	226
Included FPC contigs	321
BACs in FPC contigs	610
Total size of FPC contigs [Mbp]	48
Total number of contigs	715
Total BACs included	21,865
Total size of the backbone [Mbp]	274
Total coverage of 1AS [%]	99

The backbone consist mainly of contigs assembled with the LTC software (Frenkel *et al.,* 2010) and is complemented with BACs from an earlier assembly with the FPC software (Soderlund *et al.,* 1997, Table 1).

**Figure 2:**
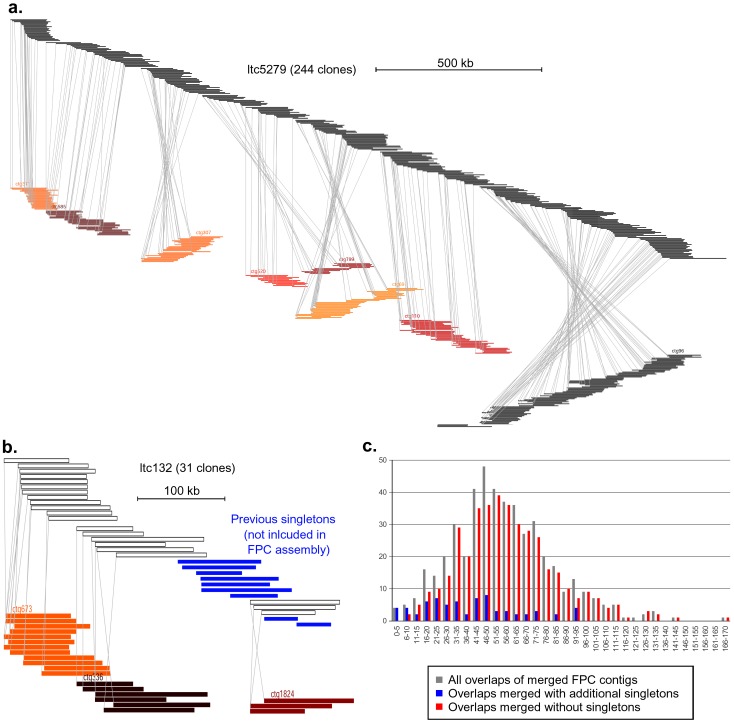
Comparison of LTC and FPC assemblies. **a.** The BAC clones constituting the contig ltc5279 are depicted at the top. Underneath, FPC contigs which cover corresponding regions are displayed. Gray lines connect the start points of corresponding BACs. Contig ltc5279 (approximately 2,161 kb in size) which is the fusion product of eight smaller FPC contigs. Overall, the relative positions of BAC clones within LTC and FPC contigs are very similar. **b.** Example of three small FPC contigs which are merged into one LTC contig (ltc132). This LTC contig also includes BACs which were singletons in the FPC assembly (blue). Note that in **a** and **b** the scales are different. **c.** Size distribution of overlaps of FPC contigs which were merged in the LTC assembly. The x-axis indicates the size range of overlaps of two FPC clones that were merged by LTC. The y-axis shows how many cases were identified in each size range. The gray series shows the size distribution of all overlaps. The blue series shows only those cases where additional singletons were included to merge FPC contigs while the red series shows the cases where no additional clones were used for the merging.

To assess the differences between the LTC and the FPC assemblies, we designed software that compares each LTC contig with the corresponding FPC contigs. As the examples in Figure 2a and 2b show, the lower number of LTC contigs and their larger size is mainly due to the merging of multiple FPC contigs into one LTC contig. In total, 208 LTC contigs are fusion products of 472 FPC contigs. In all except 6 cases, the fused FPC contigs actually overlapped but were not viewed significant by FPC. On average the merged FPC contigs overlapped by approximately 57 kb (Figure 2c). In 71 cases, the merging was done by including additional clones that were initially marked as singletons in the FPC assembly. There, the average overlap of the merged FPC contigs was 42 kb (Figure 2c). In six cases, a gap between contigs was closed entirely with FPC singletons (example in Figure 2b). These data indicate that the LTC program was more successful in producing larger, more integrated assemblies.

The reassembly of fingerprints using LTC and the comparison to the results from FPC highlighted two major problems of BAC fingerprinting in large and repetitive genomes. First, despite the use of cleaning software [34], well-to-well contamination during the HICF fingerprint sequencing can lead to many Q-clones, and in turn, non-linear Q-contigs. The fact that LTC removed these from the assembly is probably the reason why the 394 LTC contigs (despite an overall larger size of the contigs) cover only approximately 226 Mb (82% of the 1AS chromosome arm) while FPC contigs reached a 86% coverage of 1AS (Table 2). Second, the complexity of the wheat sequence limited the use of the standard physical mapping software FPC, because non-linear contigs were produced already at the initial contig construction, creating a faulty base for the map.

### The 1AS Backbone Consists of LTC BAC Contigs Complemented with FPC Clones

The final dataset of 394 LTC BAC contigs represent over 80% of the backbone of the 1AS physical map (Table 3). However, these LTC contigs do not include all BACs, which were part of the minimum tiling path produced by FPC. These excluded BACs contain potentially useful information as they were used for the NimbleGen hybridisation (see below) as well as end-sequenced. To not lose this information, we added to the above dataset of 610 BAC clones that were excluded from the LTC assembly but were part of the minimum tiling path of the FPC assembly. Of these 610 BACs, 144 were singletons while the rest were arranged in 177 small contigs of 2–7 BACs. Thus, the final backbone of the 1AS physical map consists of 394 LTC contigs with a cumulative size of 205 Mbp and 321 FPC BAC contigs or singletons that cover 50 Mbp (Table 3). The total cumulative size of all BAC contigs is 255.4 Mbp, corresponding to 92,8% of chromosome 1AS (Table 3).

In summary, we propose the use of both, LTC and FPC software. LTC overall performed better than FPC on 1AS BAC fingerprints, resulting in larger and more robust contigs. However, we also considered it important to include BAC and BAC contigs that were leftover from the initial FPC assembly. Although this leftover FPC dataset consists of BAC singletons or very short contigs, its inclusion nevertheless increased the overall coverage of the 1AS chromosome arm from 82% to almost 99%. Because these additional FPC BAC contigs have an overall low coverage and are therefore less robust than the FPC contigs. Thus, they were flagged in the final backbone with the prefix “fpc”.

### Sequence Composition of Chromosome 1AS Inferred from MTP BAC-end Sequencing

**Figure 3:**
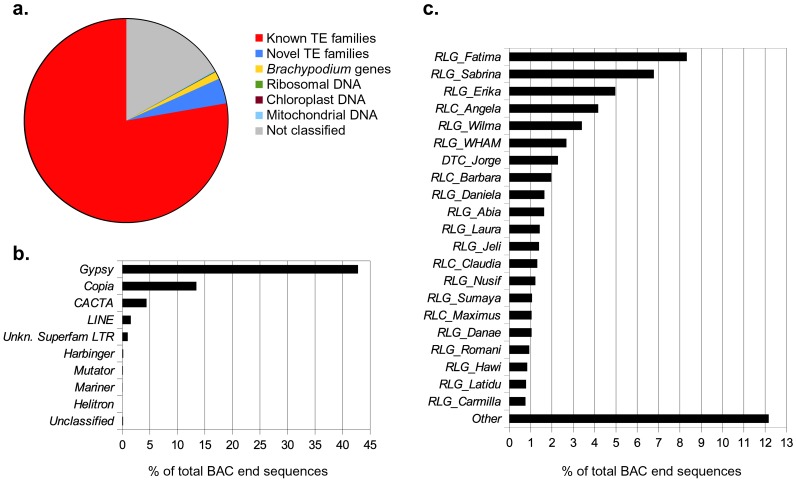
Sequence composition of minimum tiling path BAC-end sequences from chromosome 1AS. **a.** Pie chart indicating the composition of chromosome 1AS MTP BAC-ends. **b.** and **c.** Composition of the TE fraction. **b.** Composition of TE superfamilies. **c.** Composition of the TE fraction broken down into TE families. The three-letter code preceding the family name is according to the TE classification system proposed by Wicker et al., 2007.

BAC-end sequencing was performed on each of the 3,414 MTP BACs producing 6,687 sequences with an average size of 703 bp. The cumulative length of all BAC end sequences (BES) was 4,704 kb (1.7% of the 1AS chromosome arm). These sequences allowed the quantitative study of the chromosome arm sequence composition. A total of 180 sequences (2.6%) had homology with *Brachypodium* genes, 42 of which had their closest homologs in the 1AS syntenic region in the *Brachypodium* genome. These BAC ends were used to anchor BAC contigs to the *Brachypodium* reference zipper (see below). As in previous surveys of wheat BES [11], [45], a majority of sequences were identified as TEs, with 81.7% of the total DNA matching known elements (77.7%) from the TREP database, or novel TEs (4.0%) identified using protein matches to PTREP. Ribosomal and organellar DNA were present only in minuscule amounts and a total of 16.75% of all BES sequences could not be classified based on the databases used (Figure 3a).

The vast majority of TE sequences corresponded to elements of the Gypsy superfamily (42% of all BES). *Copia* and *CACTA* elements followed a distant second and third with 13.4% and 4.4% of all BES, respectively (Figure 3b). All other superfamilies, except for *LINEs* with 1.5%, contributed less than 0.2% to all BES. A total of 25.7 kb (0.54% of all BES) matched the *RLG_Cereba* elements which are *Gypsy* retrotransposons that are highly specific for centromeres [46], [47].

Interestingly, the composition of 1AS BES sequences was somewhat unexpected with regard to individual TE families. The most abundant TE family was the *Gypsy* family *Fatima,* which contributed over 8% to the total BES (Figure 3c). Similar findings were made on wheat chromosome 3B where *Fatima* was also the most abundant element [9]. In previous genome surveys of diploid Triticeae species, *Copia* elements of the *Angela/BARE1* family were clearly the most abundant with more than 10–12% of the whole genomic sequence [48]–[52]. In contrast, in our dataset, *Angela/BARE1* only contributed 4.16% to the BES. This could indicate that the TE composition of the hexaploid wheat genome differs from that of diploid Triticeae. Alternatively, it is possible that the BAC MTP does not cover the repetitive fraction of the chromosome arm evenly.

### Construction of a 1S Reference Zipper from *Brachypodium*, Rice and Sorghum Genes

Because only relatively few genetic markers were available which could be used to arrange BAC contigs into their correct physical order, we mostly used synteny (i.e. conserved order of genes) between Triticeae and sequenced model grass genomes to anchor BAC contigs. The region syntenic to wheat chromosome 1AS is located in the central part of *Brachypodium* chromosome 2 between genes *Bradi2g30410* and *Bradi2g40150* [5]. As wheat contains many TEs, we chose very stringent criteria for gene identification. First, we screened all 26,191 annotated *Brachypodium* genes for TE sequences to identify TEs that were wrongly annotated as genes. The 732 sequences that showed homology to TEs were removed from the *Brachypodium* gene set. Second, we used all the TE-filtered *Brachypodium* CDS for BLASTN searches against the rice and sorghum genomes and only used genes that have homologs at the DNA level in *Brachypodium*, rice and Sorghum. This was done to minimise the number of annotation artefacts. A total of 21,059 *Brachypodium* genes have homologs in both rice and sorghum.

Using the above described criteria, *Brachypodium* contains 600 genes which fulfilled our criteria in the 1S syntenic region. To account for genes that might have been moved out of this region in *Brachypodium*, we added (“zipped in”) those *Brachypodium* genes whose homologs are present in the group 1 syntenic region in both rice and sorghum (this principle is described in more detail by Mayer *et al.* [7]). The final 1S reference zipper contains 749 genes. To facilitate work with the reference zipper, the genes were numbered consecutively BdW1g-1 through BdW1g-749, with BdW1g-1 corresponding to the telomeric end of 1AS and BdW1g-749 to the centromere. We refer to these numbers whenever we use the term “zipper position” or “zipper gene” hereafter in the manuscript.

Compared to rice and Sorghum, *Brachypodium* contains an inversion between zipper position 127 (*Bradi2g38720*) and 280 (*Bradi2g37100*). As genetic mapping indicated, this inversion is specific to *Brachypodium* and is not found in Triticeae (our own unpublished data). Thus, for our analyses, we inverted the order of all genes between 127 and 280 in the *Brachypodium* reference zipper.

### Estimate of Gene Content of 1AS Chromosome Deletion Bins

Three chromosome deletion lines from wheat are available which lack different segments of 1AS (i.e. deletion bins). Bin 1AS-0.86–1.00 corresponds approximately to the telomeric 14% of 1AS, bin 1AS-0.47–0.86 to the region of 47%–86% of the length of 1AS and 1ASC-0.47 corresponds to the centromeric 47% of 1AS. A total of 383 1AS-specific ESTs were previously mapped to the individual bins [23], [24], 196 to the telomeric bin, 171 to the central and 16 to the centromeric bin (Table 4).

**Table 4:** Deletion bin-mapped wheat ESTs on wheat chromosome arm 1AS and estimates of gene density within the bins.

Deletion bin	size [Mbp]	ESTs^a^	Zipper hits^b^	Zipper genes^c^	gene density^d^
1AS-0.86–1.00	39	196	25	1–200	5.12
1AS-0.47–0.86	107	171	41	200–650	4.20
1ASC-0.47	129	16	1	650–749	0.77
Total	275	383	67	1–749	2.72

a Number of wheat ESTs mapped specifically to respective deletion Bin (Sorrels et al., 2003).

b Number of genes in the Brachypodium 1S reference zipper which have homology at the nucleotide level to bin-mapped ESTs.

c Approximate range of zipper genes corresponding to wheat deletion bin.

d Approximate gene density, assuming the same number of genes for wheat as in the 1AS syntenic region in *Brachypodium*.

**Figure 4:**
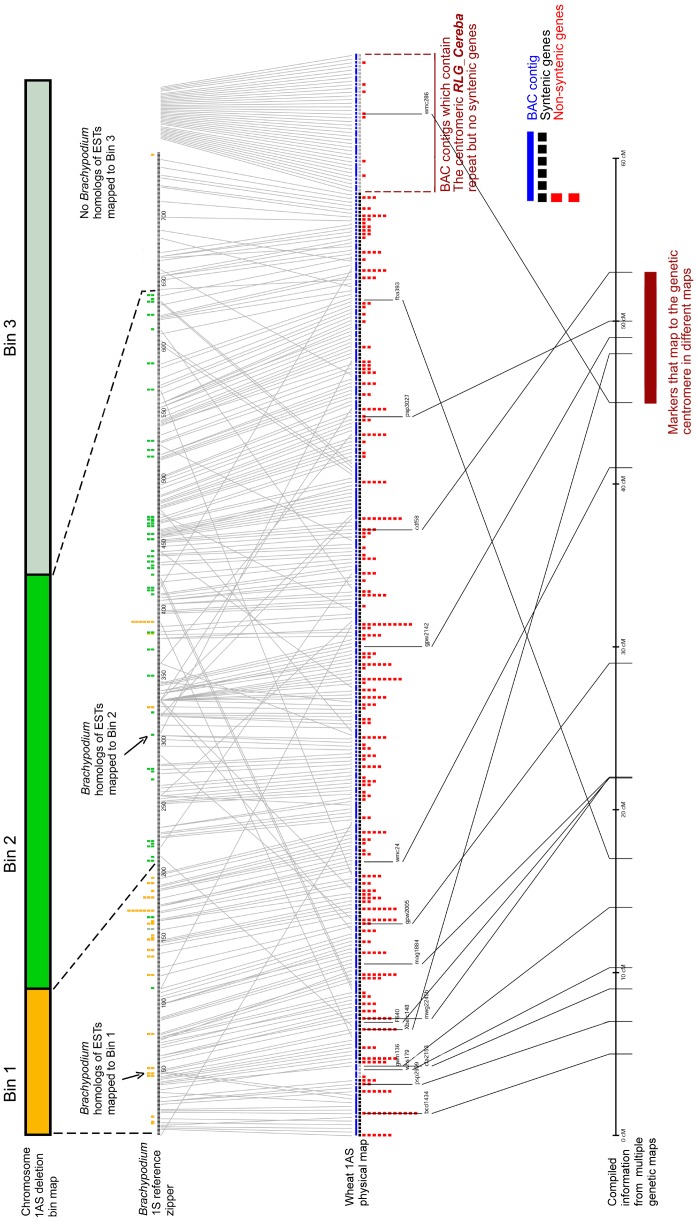
Physical map of wheat chromosome 1AS. The figure integrates multiple sequence resources. **a.** Chromosome 1AS deletion bin map with the three bins shown in (yellow, green and gray). ESTs from the three deletion bins which were mapped to *Brachypodium* reference zipper genes are indicated with boxes with colour of the corresponding bin. If more than one EST mapped to the same Brachypodium gene, the boxes were stacked on top of each other. This information was used to estimate the boundaries of each deletion bin in the *Brachypodium* reference zipper (dashed lines). **b.** *Brachypodium* reference zipper. **c.** Physical map of the 1AS chromosme arm. BAC contigs are symbolised with blue lines (see enlarged legend at the right). The length of the line reflects the number of putative syntenic genes found on the contig, not its physical size. Syntenic genes are also symbolised by black boxes. The number of non-syntenic genes for each contig is indicated with a stack of red boxes. Grey boxes indicate place holders for contigs that contained no syntenic genes but were anchored by means other than synteny (e.g. genetic markers of centromere-specific repeats. **d.** Published genetic markers from chromosome 1AS that were used to deduce an estimated genetic map (marker and map names and genetic distances are detailed in Table 1).

We used all bin-mapped ESTs in BLASTN searches against the *Brachypodium* reference zipper in order to estimate the total number of genes that are located in each of the three bins. A total of 120 ESTs showed homology at the DNA level to Brachypodium zipper genes. Often, multiple ESTs mapped to same gene and consequently, the 120 ESTs actually only represent 67 different genes (Figure 4). Nevertheless, ESTs from the telomeric and the central bin clearly mapped to separate regions of the *Brachypodium* reference zipper (Figure 4), with few exceptions, which most likely represent non-colinear genes (see below). The boundary between the telomeric and the central bin can be narrowed down to a relatively small region approximately at zipper gene 200 (Figure 4).

The boundary between the central and the centromeric bin is less clear, as only two ESTs from the centromeric bin had matches on the *Brachypodium* reference zipper and both mapped to the same gene which is located near the telomeric end of the reference zipper (Figure 4). We therefore defined the centromeric bin by the absence of EST matches and the boundary between the central and the centromeric bin to lie approximately at zipper gene 650 (Figure 4).

Assuming that the number of genes on 1AS is similar to that in the *Brachypodium* reference zipper, we estimate the telomeric bin to contain approximately 200 genes (zipper positions 1 through 200) corresponding to a gene density of about 5.1 genes per Mbp. The central bin probably contains approximately 450 genes (zipper positions 201 through 650) and thus has a density of 4.2 genes per Mbp while the centromeric bin contains approximately 100 genes (zipper positions 651 through 749) at a gene density of 0.77 genes per Mbp. These estimates should be taken with caution as they only refer to putative syntenic genes and do not include non-syntenic ones. Indeed, recent low-coverage survey sequencing of chromosome 1AS indicated the presence of a almost 1,000 non-syntenic gene sequences. However, it is suspected that many of these may be gene fragments or pseudogenes [9].

### Level 1 Anchoring: Assigning Genes to BAC Contigs Through NimbleGen Array Hybridisation

**Figure 5:**
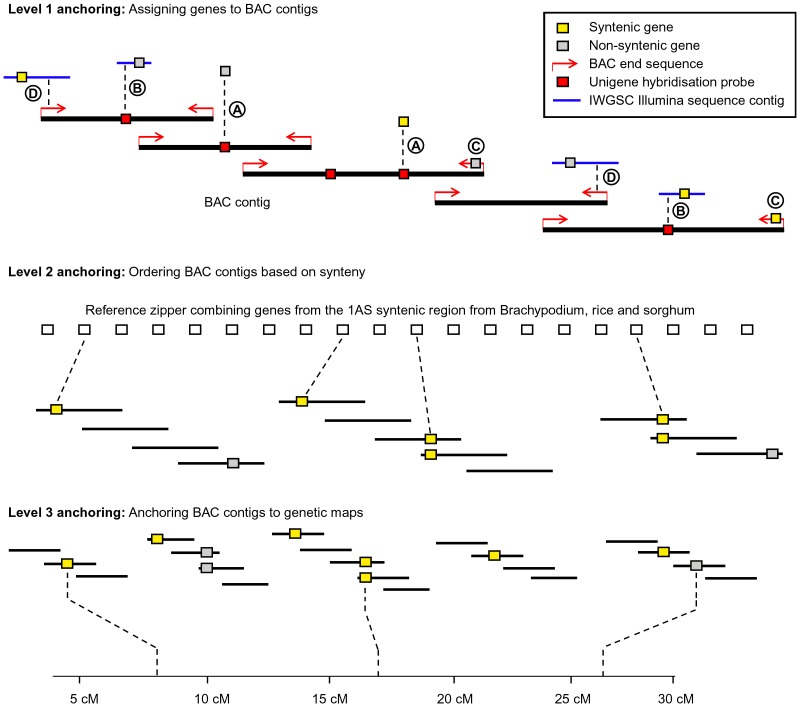
The three levels of anchoring used in the construction of the chromosome 1AS physical map. On level 1, genes were anchored to physical BAC contigs using positive hybridisation probe matches, BAC-end sequences and Illumina contigs. Individual anchoring procedures are indicated by capital letters in circles and described in the text. For level 2 anchoring, all BAC contigs which contain genes which have their homologs in the 1AS syntenic region of *Brachypodium*, rice or sorghum were anchored to the reference zipper. This means that the order of genes in wheat was assumed to be the same as in *Brachypodium*, rice or sorghum. In the final step (level 3), data from genetic markers were used to anchor BAC contigs to previously published genetic maps.

Three levels of anchoring were used to construct the integrated 1AS physical map (Figure 5). At the most basic level (level 1, Figure 5) we compiled information from different sources to assign genes or information on gene homologs to individual BAC contigs. First, we hybridised the 49 chromosome 1AS MTP pools to a NimbleGen 12×135 K microarray containing 40,349 UniGene EST clusters. After de-convolution of the data, 647 hybridisation probes with matches to MTP BAC clones were identified. Of these 647 UniGene sequences, 389 had homology to *Brachypodium* genes (Figure 5, procedure A). Because some UniGenes cover different regions of the same gene, the 389 probes actually represent only 323 different genes. Of these, 154 have homologs in the reference zipper (Table 5). Here, it should be noted that we use the term “gene sequence” or “gene” simply meaning that the sequence shows homology to a known gene either at the DNA or at the protein level. However, we do not claim that all of the identified gene sequences are intact functional genes.

**Table 5:** Numbers of genes which were assigned to 1AS BAC contigs during level 1 anchoring.

Approach	Total genes	Zipper^a^
Hybridisation	389	154
BAC-ends	180	32
Illumina/BAC-end	117	42
Illumina/unigene	61	26
PCR	0	4
Total	747	254

a Genes that have homologs in the 1AS reference zipper and which could be used for level 2 anchoring.

Overall 272 BAC contigs were identified which gave specific signals to hybridisation probes. The contigs hybridised with between 1 and 16 probes, but most hybridised with only one or two probes (Figure S1). A total of 113 contigs contained probes that have homologs in the *Brachypodium* reference zipper. These were subsequently used to anchor BAC contigs based on synteny to *Brachypodium*, rice and Sorghum (see below).

A total of 258 UniGenes have no *Brachypodium* homologs. One possible explanation is that these UniGene sequences represent genes which do not encode proteins or genes which are specific to wheat. Alternatively, it is possible that the UniGene sequence was derived from untranslated regions of the gene transcript.

### Level 1 Anchoring: Using BAC End and Illumina Chromosome Survey Sequences

To test whether some of the 258 UniGenes without *Brachypodium* homologs could be derived from untranslated parts of transcripts, we used the 258 sequences in BLASTN searches against Illumina contigs from IWGSC chromosome survey sequencing of flow sorted wheat chromosome arms. We searched for cases where we find a homolog of a *Brachypodium* gene and a homolog of the UniGenes gene sequence next to it. These could represent cases where the UniGene represents untranslated regions of a gene. We identified 61 such cases. In 26 of them, the CDS neighbouring the UniGene had homology to a gene on the *Brachypodium* reference zipper (Figure 5, procedure B).

We also used MTP BAC end sequences to anchor genes to BAC contigs (Figure 5, procedure C). As described above, we identified 180 BAC end sequences that have homology to *Brachypodium* genes, 42 of them have their closest *Brachypodium* homolog in the 1S reference zipper. We also searched the IWGSC Illumina sequence contigs for perfect matches to 1AS BAC end sequences (see methods). The goal was the identification of gene-containing Illumina sequence contigs that could be anchored to BAC end sequences. With this approach, we were able to anchor additional 117 gene-containing sequences to BAC contigs, 32 of these genes have their best homologs in the *Brachypodium* 1S reference zipper (Figure 5, procedure D).

In summary, level 1 anchoring allowed us to assign a total of 755 gene sequences to 1AS BAC contigs, 254 of them have homologs in the *Brachypodium* 1S reference zipper (Table 5) and were used subsequently in the synteny-based anchoring (level 2 anchoring).

### Level 2 Anchoring: Arranging BAC Contigs based on Synteny with Model Grasses

The 254 genes that have homologs in the 1S syntenic region in *Brachypodium*, rice and/or sorghum were used to order BAC contigs based on synteny to the three model grasses (i.e. BAC contigs containing these genes were arranged in the same order as the genes in the reference zipper). If a BAC contig contained multiple homologs to zipper genes, priority was given to the gene with the lower zipper number. For example, BAC contig LTC1712 contains homologs of *Brachypodium* genes *Bradi2g39600* (zipper position 56) and *Bradi2g39520* (zipper position 61). The contig was therefore anchored to zipper position 56. We are aware that this could contribute to some contigs being placed in the wrong location.

In a side project, we tested whether PCR probes designed based on wheat EST sequences can be used to identify BAC clones carrying those genes. Because centromeric regions are notoriously difficult to work with we focused this effort on genes that were expected to lie in the proximal region of 1AS based on synteny. We designed PCR primers for 20 genes and were successful in identifying unique BAC addresses for four of the genes (*Bradi2g30480*, *Bradi2g30850*, *Bradi2g31900* and *Bradi2g32220*), which map to positions 657, 670, 730 and 744 of the reference zipper, respectively.

In total, we were able to anchor 158 BAC contigs to the reference zipper. The 158 contigs contain in total 580 gene sequences, the 254 syntenic ones that were used for anchoring plus 326 that were non-syntenic. These numbers agree with previous findings that indicated that approximately two-thirds of the genes on 1AS are non-syntenic [8]. However, from the available data, we can not determine if these genic sequences represent functional genes or pseudogenes. The 158 anchored BAC contigs have a cumulative size of 102 Mbp, therefore representing approximately 37% of the 1AS chromosme arm (Table 6).

**Table 6:** Statistics on level 2 anchoring of BAC contigs in the 1AS physical map.

**Non-centromeric segment**	
Non-centromeric contigs	158
Non-centromeric contigs size [Mbp]	102.1
1AS fraction covered [%]	37
Syntenic genes^a^	254
Non-syntenic genes^b^	326
**Centromeric segment**	
Centromeric contigs	26
Centromeric contigs size [Mbp]	10.7
1AS fraction covered [%]	4
Genes on centromeric contigs	40
**Total 1AS map**	
Total achored contigs	185
Total contig size [Mbp]	112.8
Total 1AS fraction covered [%]	41
total genes	620

a Genes which have their closest homolog in the 1AS syntenic region of *Brachypodium*, rice and sorghum.

b Genes which have their closest homolog outside the 1AS syntenic region of *Brachypodium*, rice and sorghum.

### Level 2 Anchoring: Identification of Putative Centromeric BAC Contigs

A total of 26 BAC contigs did not contain homologs of 1AS syntenic genes but had BAC-end sequences that contain the *RLG_Cereba* LTR retrotransposon which is highly specific for centromeres in Triticeae genomes [46], [47]. Thus, these 26 contigs were added to the physical map as a separate centromere segment. As the only criterion for the placement was the presence of *RLG_Cereba* elements, the order of the BAC contigs inside the centromere segment is arbitrary. The 26 BAC contigs have a cumulative size of 10.7 Mbp which corresponds to approximately 4% of the size of the 1AS chromosome arm. A total of 40 genic sequences were identified in these 26 BAC contigs, all of which are non-syntenic (otherwise they would have been used for synteny-based anchoring).

### Level 2 Anchored BAC Contigs Cover 41% of the 1AS Chromosome Arm

The 158 BAC contigs that were anchored through synteny plus the 27 putative centromeric BAC clones have a combined length of 112.8 Mbp, thus covering approximately 41% of the 1AS chromosome arm (Table 6). In total 44.1% of the complete assembly was anchored to the map. Anchored physical contigs contain between 1 and 17 genes, with one contig (LTC-4568) containing the maximum. LTC-4568 also has the most syntenic (9) and non-syntenic genes (8). Eleven syntenic genes were duplicated (i.e. homologs to the same *Brachypodium* gene were found on more than one physical contig).

In barley, synteny-based anchoring has been shown to reflect the true gene order in at least 70% of the cases and has proven useful for marker development [7]. The high accuracy of this approach was confirmed by the data evaluated in the recent publication of a near-complete barley genome [6]. With barley being a very close relative and a model system for wheat, we expect similar accuracy for wheat chromosome 1AS and that the majority of the BAC contigs were placed in the correct linear order along the chromosome. However, using synteny-based anchoring does not allow to determine if a particular gene is placed correctly in the map. Thus, the physical map describes the probable order of genes, but the precise chromosomal location of any given gene of interest will have to be confirmed independently by genetic and/or physical mapping (e.g. radiation hybrid mapping).

### Level 3 Anchoring: Adding Genetic Markers to the Physical Map

A total of 16 genetic markers for chromosome 1AS deposited at GrainGenes could be linked to 1AS BAC-end or Illumina sequences (Table 1, see methods). These markers originate from eight different mapping projects and/or populations, two of them are composite/consensus maps (Table 1). This makes it difficult to compare and integrate specific positions on genetic maps because the same markers used in different populations can give different map positions. Eleven of the markers had been compiled in a consensus SSR map [53] and/or a composite map (Wheat-Composite2004-1A, Appels et al., unpublished, Table 1). To obtain a consensus genetic map, we calculated for each marker the average of available cM positions from different maps, being aware of the known limitations and problems of such an approach [54]. Markers and their genetic map positions relative to the telomere were then integrated with the synteny-based physical map (Figure 4). Two markers (whs179 and F640) were integrated based on local genetic maps of the *Pm3* [29] and *Lr10* loci [55], respectively.

The 16 genetic markers anchor 16 BAC contigs to a compiled genetic map of 50 cM (Figure 4). The order of the genetic maps largely corresponds to the order of the BAC contigs that was inferred from synteny to *Brachypodium*, rice and sorghum. In only two cases (*barc148* and *fba393*), the position on the genetic map contradicted the synteny-based anchoring. Synteny-based anchoring placed *barc393* close to the centromere while it genetically maps close to the telomere. The inverse situation was found for marker *barc148.* Based on synteny, *barc148* was anchored distantly at reference zipper gene 48 (*Bradi4g38230*). Further investigation showed that the gene was found in this position only in rice and Sorghum, while it is absent in *Brachypodium*. The homolog (*Bradi4g38230*) was inserted (“zipped in”, see methods) based on the rice and sorghum data. We conclude that in this case, movement of this gene in the Brachypodium/wheat lineage led to incorrect anchoring of a BAC contig.

### Level 3 Anchoring: Distal Regions Show Higher Recombination Frequencies than Proximal Ones

Interestingly, the distal regions of the transcriptome-based physical map showed a high density of genetic markers, especially in well-studied regions containing the *Pm3*, *Lrk10* and *Lr10* disease resistance loci. Indeed, there are 10 genetic markers clustered in the distal 10% of the physical map. However, on the genetic map, these 10 markers are spread out over more than 20 cM (Figure 4). Three genetic markers *psp3027*, *barc148* and *wmc286* all genetically mapped to the centromere region (Figure 4). In general, the centromere was shown to be largely recombination-free [56]. In the cases described here, the genetic centromere is spread out over almost 10 cM. This is because the centromere is at different cM position depending on the population that was used. Similarly, the 12 cM genetic distance between the *Pm3* and *Lr10* loci in the distal region of the physical map should not be taken at face value because different populations were used to map these two genes [29], [30].

Although limited, the genetic data that we could integrate in the 1AS map is nevertheless insightful and it has implications for future genetic mapping and breeding projects. Most importantly, it shows that large parts of the 1AS chromosome arm show very little recombination (at least in the genetic data that was available for this study). Previous studies on wheat chromosomes indicated that recombination frequencies are highest in distal regions while centromeric region show almost no recombination [57]. For example, on chromosome 3B, 42% of the physical map corresponds to only 2.2% of the genetic map in the centromeric regions [18]. On 1AS, approximately half of the 1AS genetic map represents only about 100–120 genes of the 1S reference zipper. This means that half of all recombination events take place in a physical region that contains less than the distal 15% of genes. Thus, different approaches such as radiation hybrid mapping that are independent of recombination [58] will be necessary to precisely map most of the wheat chromosomes.

### Evaluation of the Chromosome 1AS *Pm3* locus using Physical Contigs and Sequenced BACs

**Figure 6:**
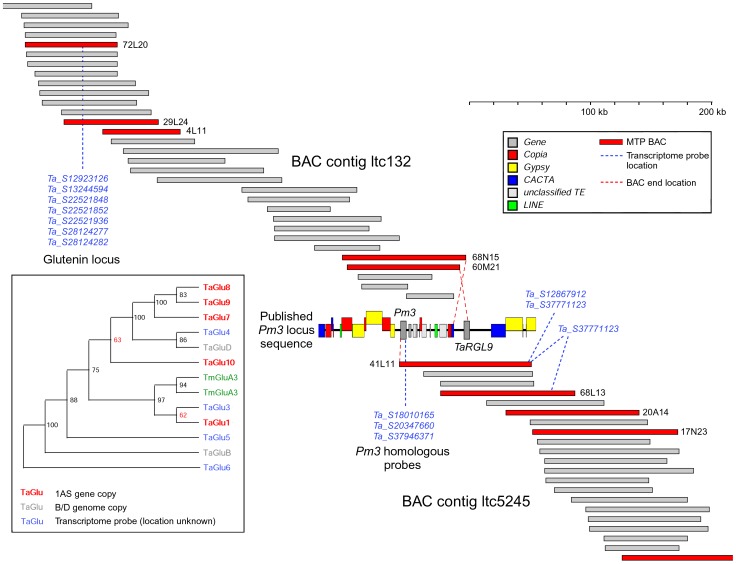
Small scale validation of the chromosome 1AS physical map using information from the previously published *Pm3* powdery mildew resistance locus. Two assembled physical contigs (ltc132 and ltc5245) were linked together using a previously published 178 kb sequence from chromosome 1AS (cv. Chinese Spring) covering the the *Pm3* locus (Wicker et al. 2007). Approximate locations of NimbleGen transcriptome hybridisation probes are shown in blue. The *Pm3* and the low molecular weight (LMW) glutenin loci are known to be closely linked (Wicker et al. 2003; Wang et al. 2010). The inset shows a phylogenetic analysis that compares glutenin UniGene sequences with previously published glutenin genes from 1AS (TmGluA3, green), 1BS (TaGluB) and 1DS (TaGluD).

To investigate the validity of the physical map at a smaller scale, we used the previously published sequence covering the *Pm3* locus (accession number AY146587 [59]). This sequence has a size of approximately 178 kb and was isolated from wheat cultivar Chinese Spring and should therefore have its exact match in the 1AS physical map. BAC end sequences and positive NimbleGen hybridisation probe sequences were used to align the published *Pm3* BAC sequence to four 1AS BAC clones (TaaCsp1AShA0068N15, TaaCsp1AShA0060M21, TaaCsp1AShA0041L11 and TaaCsp1AShA0068L13). These were placed in two different physical contigs (ltc132 and ltc5245). Interestingly, those two BAC contigs were placed directly adjacent to each other in the synteny-based anchoring (see above) and the published *Pm3* BAC sequence links these two contigs together (Figure 6).

A total of 12 UniGene hybridisation probes were found on the two physical contigs. These could be assigned to two gene loci. Three of the probes correspond to the *Pm3* gene while a second cluster of 7 probes corresponds to one or more glutenin genes (Figure 6). This is in agreement with previous studies, which reported the presence of multiple low-molecular weight glutenins at the *Pm3* locus [60], [61].

To further characterise the glutenin gene cluster, we used the 7 EST UniGene probes that mapped to the glutenin locus in Blastn searches against the IWGSC survey sequences from flow sorted chromosomes. Five of the probes had their best matches in the 1AS Illumina dataset while two originate from chromosome 1BS. Phylogenetic analysis of all probe sequences plus previously published glutenin sequences suggested that a cluster of five genes is present on BAC contig ltc132. Furthermore, using the physical mapping data available from ctg132, we estimate the glutenin gene cluster to be between 52 and 84 kb away from the left end of the contig and 100 kb away from the *Pm3* gene. Despite the rapid divergence of these loci, the estimation of 100 kb between the two gene loci compares well with the previous estimates in three different wheat ploidy levels [61].

The order of BAC-ends and NimbleGen hybridisation probe homologs on the published *Pm3* sequence corresponds nearly perfectly with the order of BACs inferred by LTC. Furthermore the close physical association of the *Pm3* gene with a glutenin cluster agrees with previously published genetic and physical mapping data [62], [63]. These findings are strong indicators for a high quality and robustness of the LTC fingerprint assembly and the 1AS physical map in general.

### Construction of a 3.8 Mbp Integrated Sequence Model

Having Illumina sequences that completely cover the 1AS chromosome arm available through IWGSC, we aimed at integrating as much of the sequence data in the 1AS physical map. As described in the methods, we used BAC-end as well as NimbleGen UniGene sequences from anchored BAC contigs to identify the matching Illumina contigs. Here it must be noted that we did not mask repetitive sequences but applied very stringent criteria for the Blastn searches. This was done to allow anchoring of possible gene sequences that are located next to repetitive sequences on an Illumina contig (example in Figure 5a, case D).

In total, we anchored 852 Illumina sequence contigs to the 185 BAC contigs that were ordered in the level 2 and level 3 anchoring steps. The 852 Illumina contigs have a cumulative size of 3.82 Mbp and therefore represent approximately 1.4% of the 1AS chromosome arm. The sequences were ordered in a single fasta flatfile according to their position in the physical map (i.e. the first sequence in the flatfile represents the one closest to the telomere). The definition line (“DE line”) of each of the 852 Illumina sequences contains basic information on the main characteristics of the sequence (Example in Table 7). The sequence model contains a total of 444 gene sequences, which are annotated with their approximate positions.

**Table 7:** Information contained in the definition lines of Illumina sequences that were used for the integrated 1AS sequence model in the order it is given in the definition line.

Name	Example	Explanation
Identifier	1AS_z_110	Chromosome arm and zipper position
Illumina name	1AS_c-5660802	Original name of Illumina contig
Gene content	Bradi3g05850 282–1376	Approximate position of gene(s)
Contig position	on ctg: 108–216	Position of respective BAC in contig [kb]
NimbleGen probe	signal = Ta_S12867912	Name of probe that gave a signal

## Conclusion

In this study we aimed at integrating multiple different resources to produce a framework that can be used for future research and mapping projects, with the ultimate goal of a complete genomic sequence of the 1AS chromosome arm of wheat. The 1AS physical map as it stands now can serve research in multiple ways. For example, it provides practical “entry points” for genetic mapping projects. The anchored BAC-end and Illumina sequences can serve for the development of new genetic markers. Here, resources from 454 whole-genome sequencing [62] can be used to produce markers based on single nucleotide polymorphisms (SNPs). Once a target gene is genetically located, BAC contigs can provide direct access to candidate gene sequences. Furthermore, we were able to assign almost 11 Mbp of BAC contigs to the centromeric region of 1AS. This is noteworthy as many important genes are located in the centromeres of wheat chromosome such as *Ph1* [63]. The map also allows the reverse approach of identifying homologous regions of important chromosome 1AS genes in model systems, providing more efficient strategies in studying gene function [64], [65].

To further advance the 1AS physical mapping project, it will be of great importance to integrate a much larger number of genetic markers. This will allow to move away from ordering of sequences based on synteny to model genomes to inferring sequence order based on actual wheat data. Next generation sequencing of sorted chromosome arms from different wheat genotypes can be used as a source of genetic markers to accomplish this task in the near future [66]. Additionally with the decreasing costs of sequencing, a complete sequencing of the 1AS minimum tiling path in now conceivable and will be an essential step toward the ultimate goal of a complete genomic sequence of the 1AS chromosome arm of wheat.

## Supporting Information

Figure S1 **Number and distribution of UniGene probes that hybridised to BAC contigs.** The x-axis indicates the number of UniGene probes per BAC contig. The y-axis indicates how many BAC contigs contain the respective number of probes (e.g. 75 BAC contigs contain produced hybridisation signals to exactly one UniGene probe). In total, 272 BAC contigs contain UniGene probes.
(PDF)
