# Supplementary material for: A Physical Map of the Short Arm of Wheat Chromosome 1A
Source: PLoS One. 2013 Nov 21;8(11):e80272. doi: 10.1371/journal.pone.0080272 (PMC3836966; doi:10.1371/journal.pone.0080272)
Supplement: Figure S1 — Number and distribution of UniGene probes that hybridised to BAC contigs. The x-axis indicates the number of UniGene probes per BAC contig. The y-axis indicates how many BAC contigs contain the respective number of probes (e.g. 75 BAC contigs contain produced hybridisation signals to exactly one UniGene probe). In total, 272 BAC contigs contain UniGene probes. (PDF) [file pone.0080272.s001.pdf]

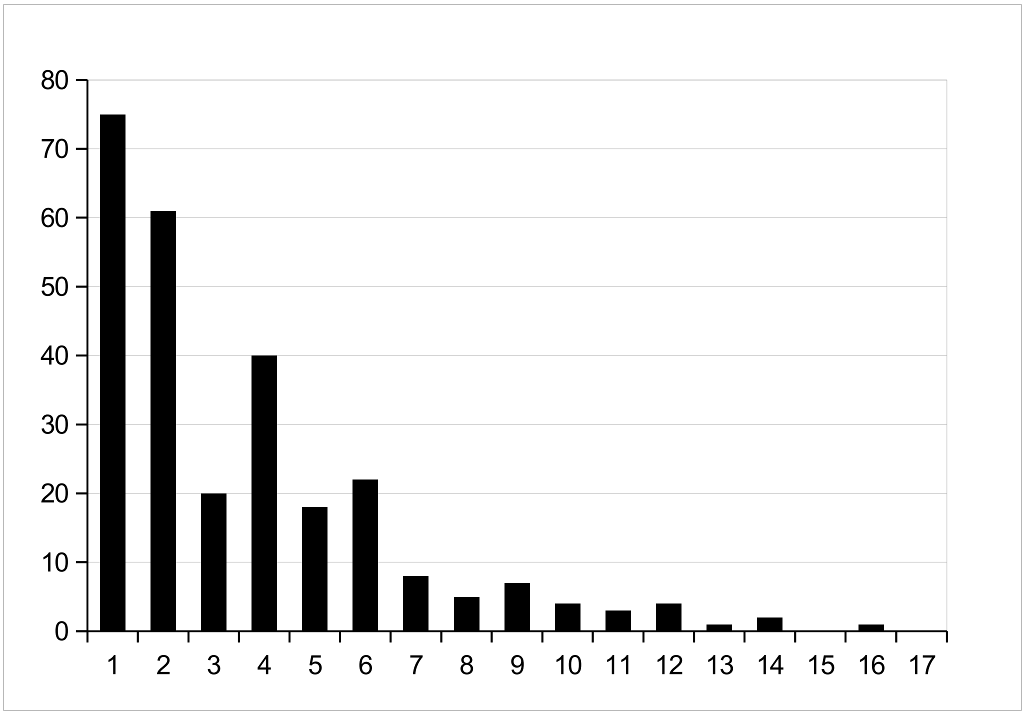

Figure S1. Number and distribution of UniGene probes that hybridised to BAC contigs. The x-axis indicates the number of UniGene probes per BAC contig. The y-axis indicates how many BAC contigs contain contain the respective number of probes (e.g. 75 BAC contigs contain produced hybridisation signals to exactly one UniGene probe). In total, 272 BAC contigs contain UniGene probes.
